# Intravitreal injection of low-dose gentamicin for the treatment of recurrent or persistent uveitis in horses: Preliminary results.

**DOI:** 10.1186/s12917-018-1722-7

**Published:** 2019-01-16

**Authors:** Britta M. Fischer, Richard J. McMullen, Sven Reese, Walter Brehm

**Affiliations:** 1 Department of Clinical Sciences, College of Veterinary Medicine, Auburn University, JT Vaughan Large Animal Teaching Hospital, 1500 Wire Road, Auburn, AL 36849-5540 USA; 2 Chair of Anatomy, Histology and Embryology, Faculty of Veterinary Medicine, LMU, Munich, Germany; 3 Faculty of Veterinary Medicine, University of Leipzig, Department for Horses, Leipzig, Germany

**Keywords:** Equine recurrent uveitis, Gentamicin, Intravitreal injection, Equine ophthalmology, *Leptospira*

## Abstract

**Background:**

Despite appropriate medical therapy, many horses with equine recurrent uveitis continue to suffer from recurrent bouts of inflammation. Surgical intervention via the pars plana vitrectomy or suprachoroidal cyclosporine implant placement may control and/or prevent recurrences, however, these procedures may be contraindicated, unavailable, or declined by an owner. Thus, an effective adjunctive treatment option may help to improve the clinical outcomes in those situations. There are several anecdotal reports on the use of intravitreal gentamicin injections, but to date, no data evaluating the complication rate and/or treatment effect following this treatment have been published. Thus, the aim of this prospective study was to describe the intravitreal gentamicin injection technique, describe the associated peri-injection (within 24 h) and post-injection (30 to 780 days) complications, and to report the effects of the injection on the clinical signs of uveitis. Additionally, evaluation of the systemic and ocular *Leptospira*-status, and its effect on the treatment outcome was performed. A total of 86 horses of various ages, breeds, and gender presenting with recurrent or persistent uveitis were treated via intravitreal injection of 4 mg of undiluted gentamicin (0.04 ml, Genta 100, 100 mg/ml in 35 horses) or preservative-free gentamicin (0.05 ml, 80 mg/ml in 52 horses) under sedation and local anesthesia. All 86 horses were observed for immediate peri-injection and post-injection complications. Response to therapy was evaluated in 59 of the 86 horses (follow-up: 30 to 780 days).

**Results:**

Peri-injection complications consisted of subconjunctival (26/86; 30.2%) or intracameral hemorrhage (4/86; 4.7%); both of which completely resolved within 5 days. Post-injection complications consisted of cataract formation/maturation (5/59 horses, 8.5%) and diffuse retinal degeneration (3/59 eyes 5.1%). The majority of horses 52/59 (88.1%) with a minimum follow-up period of 30 days were controlled (absence of recurrent or persistent inflammation) at their last recheck examination. Recurrent inflammation was documented in 5/59 (8.5%) horses and persistent inflammation was diagnosed in 2/59 (3.4%) horses.

**Conclusions:**

Intravitreal injection of low-dose gentamicin shows promise at controlling different types and stages of uveitis. The ability of intravitreal injections of low-dose gentamicin (4 mg) to control persistent and recurrent inflammation warrants further investigation.

## Background

Equine recurrent uveitis (ERU) is widely recognized as an immune-mediated disease characterized either by recurrent bouts of ocular inflammation separated by variable periods of quiescence (lack of detectable ocular signs associated with active inflammation) or low-grade, persistent inflammation [1–3]. The cornerstone of treatment for ERU consists of local immunosuppression or immune-modulation in conjunction with systemic anti-inflammatory treatment [4–7].

In addition to medical therapy, there are two widely utilized surgical procedures, cyclosporine suprachoroidal implants (CSI) and pars plana vitrectomy (PPV), that are routinely performed to treat horses with ERU [8–14]. Implantation of a CSI has been proven to be an effective means of controlling uveitis in horses responsive to prior medical therapy [3, 8, 13, 15]. However, because their legal importation into Europe is restricted to academic institutions for specific use in ongoing research, the use of CSI is severely limited on this continent. A more commonly performed surgery in Europe (especially Germany) is the PPV [9, 10, 14, 16]. Initially, PPV was utilized to treat all forms of ERU, but recently published data suggests that it is most effective in horses with confirmed leptospiral etiology [14].

Two recent studies demonstrated the relative inability of medical therapy to adequately control and prevent long-term complications and blindness in a large proportion of horses evaluated, highlighting the importance of additional treatment modalities [17, 18]. Intravitreal injections with triamcinolone acetonide or rapamycin have been successfully utilized in the management of recurrent uveitis in humans, as well as in small groups of horses [19–23]. However, the rate of complications and lack of long-term control of ERU, has limited their use in equine ophthalmology. Gentamicin (0.2–0.4 mg/ml), a bactericidal aminoglycoside antibiotic, has been routinely added to the PPV irrigation solution since the surgery’s introduction in the early 1990s [9–11, 24]. This led to speculation that low-dose intravitreal gentamicin (4 mg) injections (IVGI) alone could serve as an alternative treatment for ERU, and initial results were presented by Pinard, et al. in 2005 [25]. Despite widespread anecdotal use, there are no published studies evaluating the efficacy of this treatment or establishing the risk of complications following IVGI.

The purpose of this prospective study was to describe the intravitreal gentamicin injection technique, to identify any peri-injection (within 24 h) and/or post-injection (30 to 780 days follow-up) complications associated with the IVGI and to evaluate the clinical outcome in horses with ERU, following a single 4 mg IVGI. Additionally, aqueous humor (AH) and serum (S) samples were evaluated for the presence of leptospiral antibody titers (S and AH) and leptospiral DNA (AH) and their effect on the treatment outcome.

## Results

### Horses

A total of 86 horses with a mean follow-up period of 165.9 ± 190.3 days (range: 1 to 780 days) were included in the present study. The mean age was 11.6 ± 5.5 years (range: 2 to 28 years). Gender, breed and coat color distribution can be found in Table 1. Twenty-nine horses were treated bilaterally, resulting in one eye being randomly selected for evaluation.

**Table 1:** Gender, breed and coat color distribution of the horses (*n* = 86) that had undergone IVGI between January 2013 – June 2016

Gender *n* = 86	Geldings	49	(57%)
	Mares	31	(36%)
	Stallions	6	(7%)
**Breed n = 86**	Warmblood	38	(44.2%)
	Quarter Horse; Paint Horse	9	(10.5%)
	Icelandic Horse	6	(7.0%)
	Pony	6	(7.0%)
	Heavy Warmblood	5	(5.8%)
	Standardbred Trotter	5	(5.8%)
	Haflinger	5	(5.8%)
	Appaloosa	4	(4.7%)
	Spanish	3	(3.5%)
	Knabstrupper	3	(3.5%)
	Thoroughbred	2	(2.3%)
**Coat Color Distribution n = 86**	Bay	36	(41.9%)
	Chestnut	16	(18.9%)
	Gray	11	(12.8%)
	Leopard-patterned	11	(12.8%)
	Black	7	(8.1%)
	Dun	5	(5.8%)

**Fig. 1:**
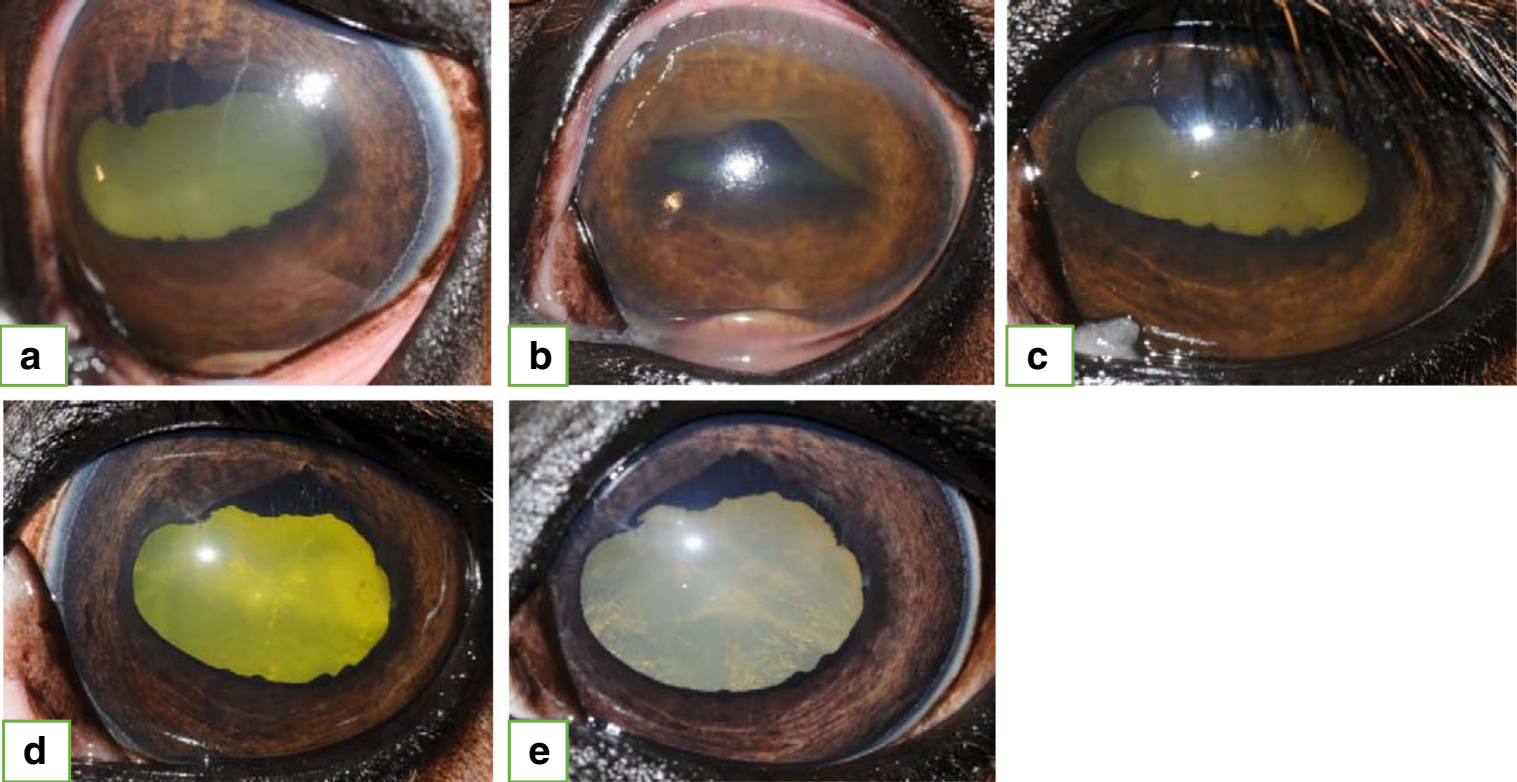
Twelve-year old warmblood mare that presented for a chronic-acute persistent panuveitis of the left eye (OS). Negative c-value for *Leptospira*. **a.** Initial presentation: Diffuse corneal edema, keratic precipitates, + 2/4 flare, fibrin, vitreal degeneration and retinal folds were present. Medical treatment was started (prednisolone acetate q 4–6 h, atropine q 8 h and flunixine meglumine 1.1 mg/kg twice daily). **b.** Ten days post initial presentation: The clinical signs worsened in the face of aggressive medical treatment. Intravitreal gentamicin injection and aqueocentesis were performed OS. **c.** Four days post-IVGI OS: Improvement of clinical signs can be readily appreciated. **d.** Twenty days post-IVGI OS: No flare present. **e.** Forty-nine days post-IVGI OS: Uveitis remains controlled without medications. The mare was euthanized due to complications associated with cervical spinal fracture 2 months after IVGI

Fifty-nine of the 86 eyes had a minimum follow-up period of 30 days (range: 30 to 780 days) and comprised the group undergoing statistical evaluation of post-injection complications and clinical treatment outcome. Fifty-two of 59 eyes (88.1%) were controlled (non-recurrence/persistence, independent of complications) after the IVGI, and despite the discontinuation of topical and medical therapy. Overall, 5/59 eyes (8.5%) presented with recurrent and 2/59 eyes (3.4%) presented with persistent inflammation during follow-up examination. The follow-up data and corresponding results of positive outcome are listed in Table 2. Category distributions are listed in Fig. 1, Tables 3 and 4.

**Table 2:** Follow-up periods and clinical outcomes post-intravitreal gentamicin injections

Minimum follow-up period	Controlled ERU (no recurrent or persistent inflammation, independent of complications)	Average follow-up period (days)	Standard deviation (± days)	Range (days)
**30 days** 59/86 (68.6%) eyes	52/59 eyes 88.1%	238	190	30–780
**3 month** 43/86 (50.0%) eyes	36/43 eyes 83.7%	313	175	93–780
**5 month** 34/86 (39.5%) eyes	27/34 eyes 79.4%	359	164	153–780
**7 month** 24/86 (27.9%) eyes	20/24 83.3%	407	170	213–780
**1 year** 12/86 (14.0%) eyes	9/12 eyes 75%	541	137	365–780

**Table 3:** Characteristics of uveitis

acute/chronic n = 86 eyes	acute 4 (4.7%)	chronic 25 (29.1%)		chronic-acute 57 (66.3%)	
**recurrent/persistent n = 86 eyes**	**recurrent** 36 (41.9%)		**persistent** 50 (58.1%)		
**presence of aqueous flare n = 86 eyes**	**0** (no flare)42 (48.8%)	**1** (faint flare) 20 (23.3%)	**2** (moderate flare) 13 (15.1%)	**3** (severe flare) 3 (3.5%)	**4** (blood or fibrin in anterior chamber) 8 (9.3%)

**Table 4:** Subjective visual assessment pre- and post-IVGI and the subsequent change, or lack thereof, in the individual eyes evaluated

Pre-IVGI n = 86 eyes	GOOD 42 (48.8%)	REDUCED 20 (23.3%)	POOR 24 (27.9%)
**Post-IVGI*n*** **= 59 eyes**	**GOOD** 38 (64.4%)	**REDUCED** 8 (13.6%)	**POOR** 13 (22.0%)
**Change in status following IVGI n = 59 eyes**	**UNCHANGED** 42 (71.2%)	IMPROVED* 11 (18.6%)	DETERIORATED** 6 (10.2%)

*from a **POOR** to a **REDUCED** or **GOOD** vision status or from a **REDUCED** to a **GOOD** vision status)**from a **GOOD** to **REDUCED** or **POOR** or from **REDUCED** to **POOR** vision status due to cataract formation or phthisis bulbi

### *Leptospira* status

Aqueous humor was obtained from 79/86 (91.9%) eyes and serum from 80/86 (93%) horses. Table 5 shows the calculated c-values for each individual *Leptospira* serovar and the combined results are documented in Table 6. *Leptospira* PCR was performed on 79 eyes, 23 of which were positive 23/79 (29.1%). Based on our inclusion criteria and calculation of c-values for each eye 50/79 eyes (63.3%) were classified as *Leptospira* negative, 13/79 eyes (16.5%) were classified as *Leptospira* suspicious and the remaining 16/79 eyes (20.3%) were classified as *Leptospira* positive.

**Table 5:** Single c-value results for individual *Leptospira* serovars

Result	C-values for individual Leptospira (L.) serovars								
	sejroe (*n* = 63)	saxkoebing (n = 63)	canicola (*n* = 63)	autum-nalis (*n* = 79)	grippo-typhosa (*n* = 79)	pomona (*n* = 79)	australis (n = 79)	ictero-haemorrhagiae/copenhageni (n = 79)	bratislava (n = 79)
**Posititive (+)**	**0**	**0**	**0**	**1** (1.3%)	**15** (19.0%)	**4** (5.1%)	**1** (1.3%)	**1** (1.3%)	**1** (1.3%)
**Negative (−)**	**63** (100%)	**62** (98.4%)	**63** (100%)	**74** (93.7%)	**54** (68.4%)	**69** (87.3%)	**75** (95.0%))	**77** (95.0%)	**75** (95.0%)
**Suspicious (?)**	**0**	**1** (1.6%)	**0**	**4** (5.1%)	**10** (12.7%)	**6** (7.6%)	**3** (3.8%)	**1** (1.3%)	**4** (5.1%)

**Table 6:** Combined serum and aqueous humor *Leptospira* titer results and the corresponding *Leptospira* c-value results

Titer	negative titer(s)	SINGLE SEROVAR positive titer (< 1:400)	MULTIPLE SEROVARS positive titers (1:100–1:400)	SINGLE SEROVAR positive titer (> 1:400)	MULTIPLE SEROVARS positive titers (> 1:400)
**Serum (*n*** **= 80)**	**41** (51.3%)	**20** (25.0%)	**10** (12.5%)	**6** (7.5%)	**3** (3.8%)
**Aqueous humor (n = 79)**	**49** (62.0%)	**11** (13.9%)	**1** (1.3%)	**13** (16.5%)	**5** (6.3%)
**C-value(different serovars)**	**NO positive c-value**	**SINGLE positive c-value** (less than 4)	**MULTIPLE positive c-values** (less than 4)	**SINGLE positive c-value** (greater than 4)	**MULTIPLE positive c-values** (greater than 4)
**C-value (n = 79)**	**50** (63.3%)	**12** (15.2%)	**1** (1.3%)	**11** (13.9%)	**5** (6.3%)

### Peri-injection complications

Subconjunctival and intracameral hemorrhage (due to the aqueous paracentesis) were seen in 26/86 (30.2%) and 4/86 (4.7%) of the eyes, respectively, but were completely resolved within 5 days.

### Post-injection complications

**Fig. 2:**
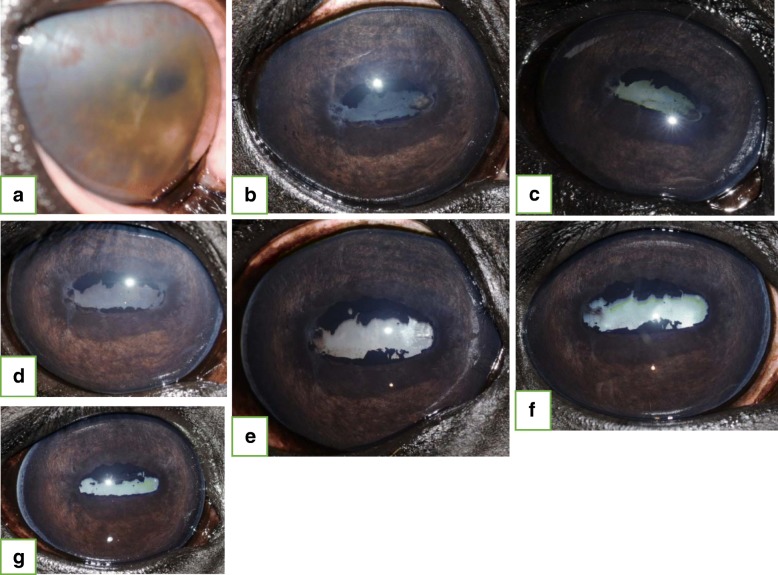
Nine-year old warmblood gelding presented with chronic-acute, persistent panuveitis of the right eye (OD). Negative c-value for *Leptospira*. **a**. Initial presentation: Blepharospasm, epiphora, diffuse corneal edema, 360° corneal neovascularization, + 4/4 flare, fibrin in anterior chamber and miosis were present. The posterior aspect of the eye could not be visualized. Inflammation was controlled with medical therapy. **b.** One-hundred-fifteen days post-initial presentation: Two additional bouts of inflammation since initial presentation. Both intravitreal gentamicin injection (IVGI) and aqueocentesis were performed. Immature cataract and posterior synechia were present at the time of IVGI OD. **c**. Seven days post-IVGI OD: Immature cataract. **d.** Sixty days post-IVGI OD: Medical treatment was discontinued 39 days prior to this examination. No signs of active inflammation could be identified. **e.** One-hundred-thirteen days post IVGI OD: Mature cataract (cataract maturation). No signs of active inflammation. **f.** Three-hundred-twenty-seven days post-IVGI OD: Uveitis remains controlled without medical treatment. **g.** Six-hundred-two days post-IVGI OD: No recurrent bouts of inflammation since IVGI

**Fig. 3:**
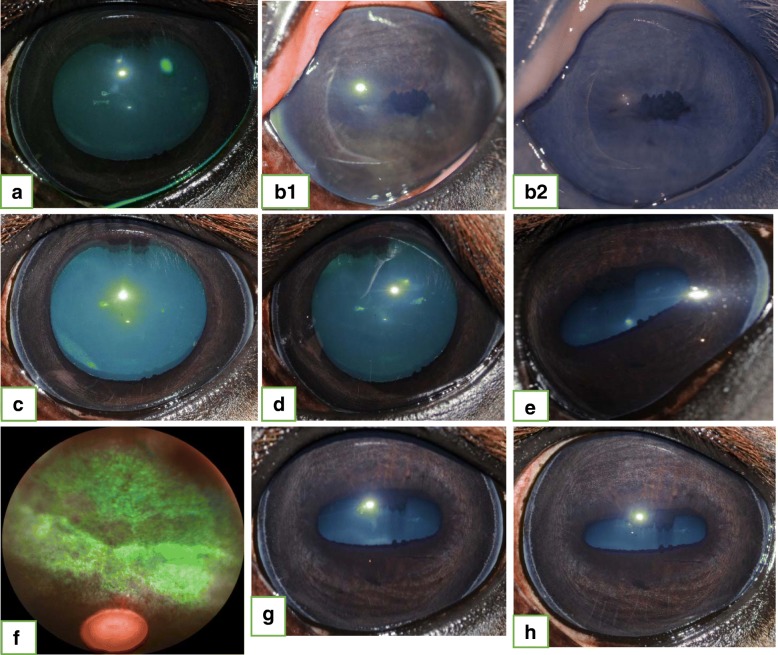
Eight-year old warmblood mare that presented for chronic-acute, recurrent anterior uveitis in the left eye (OS). Negative c-value for *Leptospira*. **a.** Initial presentation: Two days following the onset of an acute bout of inflammation. Topical and systemic therapy were initiated by the referring veterinarian, when ocular signs were first identified. Inflammation was controlled via medical therapy. **b1.** Seven months after initial presentation: Active uveitis OS; + 4/4 flare, fibrin and complete miosis were present. **b2.** Infrared picture of OS at the same examination as in B1. **c.** Uveitis was controlled within 5 days of initiating medical therapy. **d.** Recurrent acute inflammation: 14 months later. Intravitreal gentamicin injection (IVGI) and aqueocentesis were performed. **e.** Ninety-eight days post-IVGI OS: Uveitis has remained controlled following IVGI. A focal area of tapetal hyperreflectivity identified during indirect ophthalmoscopy of the fundus. **f.** Fundus image of the lesion described in **e.** Retinal degeneration developed between the 30- and 98- day recheck examination. Subjective vision status unchanged from pre-IVGI. **g.** Two-hundred-seventy days post-IVGI OS: Uveitis controlled. Retinal degeneration remains static. **h.** Three-hundred-eighty-five days post-IVGI OS: Uveitis remains controlled. Retinal degeneration remains static

Fifty-nine of 86 eyes had a minimum follow-up period of 30 days (30–780 days) and were evaluated for the presence of post-injection complications. Cataract formation/maturation was observed in 5/59 (8.5%) eyes, and retinal degeneration was seen in 3/59 eyes (5.1%). Four of the five cataracts that developed post-injection were identified in horses that received gentamicin with preservatives. Cataract progression/maturation occurred within one week (1/5, 20%), within one year (3/5, 60%), and later than one year post-IVGI (1/5, 20%). All eyes, that developed mature cataracts, presented with cataracts of different stages before IVGI (Fig. 2). Retinal degeneration, not associated with obvious visual deficits (as assessed via menace response), was identified in 3/59 (5.1%) eyes (Fig. 3), and was consistently identified as a horizontal, geographic area (between one and three disc diameters in size) of diffuse tapetal hyperreflectivity superior to the optic nerve head. This complication was identified in a single eye at each of the following time points: Within 30 days, between 30 and 60 days, and between 90 and 122 days following IVGI, respectively. None of these eyes showed signs of retinal degeneration prior to IVGI.

### Statistical evaluation of factors influencing treatment outcomes and complication rates

Variables with a significant effect on treatment outcome or on the development of long-term complications, are presented in Table 7. A significant correlation was identified between the Appaloosa breed and recurrence of inflammation (*P* < 0.001). In each of these horses, uveitis remained controlled in the early stages of follow-up. However, over time, aqueous flare was detectable in all three eyes. Leopard-patterned horses were more likely to develop recurrent inflammation than horses with other coat colors (*P* = 0.049). None of the eyes, that developed retinal degeneration, had detectable aqueous flare pre-IVGI (*P* = 0.046). The presence of subconjunctival or intracameral hemorrhage post-IVGI did not have a significant influence on either the control of the uveitis, or the development of long term-complications. Neither the clinical diagnosis, nor the additional categories utilized to subjectively grade equine uveitis in the present study had any influence on the control of the uveitis or the development of post-injection complications. The *Leptospira* antibody status of the eye (positive, suspicious, or negative C-value) and aqueous humor *Leptospira* PCR results did not have a significant influence on the development of long-term complications. There was a significant influence on the development of persistent inflammation in one eye with multiple positive C-values ≥4 (C-value of 8 for *L. pomona* and C-Value of 4 for *L. grippotyphosa*) (*P* = 0.015), multiple aqueous humor titers ≥1:400 (aqueous humor titer for *L. Pomona* 1:800 and for *L. grippotyphosa* 1:1600) (*P* = 0.013) and one positive serum titer ≥1:400 (serum titer for *L. grippotyphosa* 1:400) (*P* = 0.047). Although not significant, 4/5 (80%) mature cataracts developed following IVGI injection with gentamicin containing preservatives (Genta100).

**Table 7:** Individual variables with positive correlation to treatment outcome or the development of post-injection complications

Variable	Treatment Outcome	Post-Injection Complications
Breed	Appaloosas: more recurrent inflammation than other breeds	NC
Coat color	Leopard patterned: more recurrent inflammation than other coat colours	NC
Flare pre-IVGI	NC	NO flare: increased risk of retinal degeneration
*Leptospira* status of the eye	Multiple positive C-values (≥4) for multiple individual serovars: increased incidence of persistent inflammation	NC
Aqeuous humor titer in total	Multiple positive individual aqueous humor titers for *Leptospira* (≥1:400): increased incidence of persistent inflammation	NC
Single serum titer	One positive serum titer for *Leptospira* (≥1:400): increased incidence of persistent inflammation	NC

Abbreviations: *NC* no correlation

## Discussion

Many horses with ERU require additional treatment modalities in addition to medical therapy. Both the PPV and CSI placement are commonly performed in Europe and the USA, respectively [8, 15, 16, 26]. Although CSI placement can effectively suppress intraocular inflammation and prevent recurrent inflammatory bouts for several years, they are not readily available in Europe due to legal restrictions governing their importation. Additionally, there are several instances where PPV (e.g., radial retinal detachments, late immature to mature cataracts present) or CSI (e.g., uncontrollable inflammation despite appropriate medical therapy) are contraindicated. Thus, additional or alternative treatment options, such as IVGI, may enable us to better control this highly debilitating disease. attractive.

Aqueous paracentesis and IVGI can be performed during the same sedation, using minimal regional and topical anesthesia; thus, negating the need for general anesthesia. Ultimately, only those horses not being controlled with IVGI require additional surgical intervention. This has dramatically reduced the number of horses requiring surgical intervention to control ERU in our clinic population. However, if an eye fails to be controlled with IVGI, the *Leptopsira* status, previously obtained via aqueous paracentesis, can then be used to choose the most appropriate surgical intervention, e.g., a PPV or CSI.

Serum *Leptospira* antibodies are able to effectively cross the blood-ocular barriers in the presence of uveitis, therefore only local antibody production at the site of the inflamed tissue is a true indicator for a *Leptospira*-induced mechanism of action. Individual aqueous humor or serum antibody titers are unreliable predictors of involvement [27]. In order to accurately identify *Leptospira’s* role in the pathogenesis of equine uveitis it is important to calculate the c-value (i.e., the ratio between aqueous humor and serum antibody titers) [14].

The main goals in treating ERU are the reduction of ocular inflammation, the reduction or elimination of pain or discomfort, and the preservation of vision [3, 17]. According to a recent study by Gerding and Gilger, nearly half of the eyes affected with uveitis became blind, regardless of the therapy implemented [17]. In a study from Germany, evaluating the post-operative results following PPV for the treatment of ERU, 17/43 (39.5%) of the eyes had improved vision, 14/43 (32.6%) of the eyes demonstrated reduced vision, and 12/43 (27.9%) of the eyes were blind following the surgery [12]. Long-term results following implantation of a CSI revealed that 119/151 (78.8%) of the eyes remained visual [13]. Although the results of the present study (Table 4) are not directly comparable with the previous reports, the vision status of the eyes in this study remained unchanged following IVGI in 71.2% (42/59) eyes, were improved in 18.6% (11/59) eyes, and deteriorated in 10.2% (6/59) eyes. Each eye that developed mature cataracts post-IVGI had some degree of immature cataract maturation prior to IVGI (Fig. 2). Despite this, the degree of cataract maturation prior to IVGI cannot be reliably utilized to predict the likelihood of cataract progression/maturation following IVGI. None of the horses that developed post-IVGI retinal degeneration demonstrated any subjective behavioral changes (e.g., head carriage abnormalities (head tilt), spooky or erratic behavior, hesitant to move or navigate obstacles in a known environment) suggesting that vision was compromised, nor did they not show detectable clinical signs of vision loss (menace response). However, we cannot conclude that vision was not compromised. Additional functional testing methods, such as pre-IVGI and post-IVGI electroretinography (ERG) would provide more objective and meaningful results pertaining to retinal function, and should be considered for future studies. Although none of the three eyes that developed retinal degeneration had flare at the time of IVGI, the clinical disease progression in each of these horses differed significantly enough to prevent us from drawing a reliable conclusion as to why this complication occurred. We cannot exclude that eyes presenting with a mature cataract did not develop retinal degeneration. The risks of potential cataract maturation and retinal degeneration cannot be ignored, and must be discussed in detail with the owner when discussing treatment options. There are many factors that will ultimately determine if an IVGI is indicated, thus, it is important to provide an accurate risk-benefit analysis for each individual horse. Further investigation into the post-injection development of retinal degeneration is warranted. Ongoing efforts include functional testing via ERG and posterior segment evaluation via optical coherence tomography (OCT). The fact that there was a significant influence on the development of persistent inflammation in one eye with multiple positive C-values ≥4 of different *Leptospira* titers, multiple aqueous humor *Leptospira* titers ≥1:400 of different serovars and one positive serum *Leptospira* titer ≥1:400 warrants further investigation, but caution must be taken when interpreting this data, as only a single eye was affected. The complication rates associated with IVGI (88.1% non-recurrence/non-persistence rate, 8.5% cataract progression/maturation, and 5.1% retinal degeneration) are comparable to published results following CSI placement (46% non-recurrence rate, 16% cataract progression/formation and 16% retinal degeneration) and PPV (73.6–100% non-recurrence rate, 38.2–44.2% cataract progression/formation, and 9.3% retinal degeneration) [9–14].

Presently, the mechanism of action of gentamicin on the disease process in ERU and other types or stages of equine uveitis remains enigmatic. Positive suppression of inflammation, which can be observed as early as 24–48 h post-IVGI, was achieved in various types and stages of equine uveitis despite the *Leptospira* status of the eye, in the present study. Therefore, we speculate that rather than having a direct bactericidal effect on putative bacterial organisms, gentamicin instead influences or interferes with the immune-mediated processes intrinsic to ERU. Although purely speculative, the underlying mechanism of action of gentamicin may block or suppress the activation of specific T-cell lines; cells that are known to play a significant role in autoimmune uveitis [28]. Further research into gentamicin’s mechanism of action following intravitreal injection is necessary.

Limitations of the present study are the short follow-up periods utilized for evaluation following IVGI. Despite the short follow-up duration, a 30-day minimum follow-up period was selected in order to capture the immediate effects of IVGI and to ensure that all complications seen associated with this technique were observed and recorded. Had we selected a longer minimum follow-up period, 1/3 (33.3%) of the eyes that developed retinal degeneration and 1/5 (20%) of those, where cataract progression was observed would have not been included in our results, thus introducing a false positive bias into our complication rates. One cataract, that would not have been included otherwise, developed when using preservative-free gentamicin. Thus, setting the minimum-follow-up period at a time point further out from the IVGI (e.g., 5 months) would have prevented inclusion of those complications from our results, as a result, an incorrect conclusion would have been drawn that no cataract progression/maturation occurred when utilizing PFG for the IVGI. Future studies evaluating the clinical outcome and the presence or development of long-term complications over multiple years are necessary and are currently underway.

The present study reports control of ERU in 88.1% (non-recurrence/non-persistence rate) in the absence of medical treatment of the eyes with a minimum follow-up period of 30 days. These results support the anecdotal findings reported by Pinard in 2005 with a positive outcome of 94.4%, and those reported by Kleinpeter in 2014 showing a positive outcome in 93.3% (follow-up: 2–96 month) [25, 29]. In the latter study, 11/60 (18.3%) eyes became blind due to cataract formation following IVGI [25]. When comparing this result to the first 34 eyes in the present study that were treated with gentamicin containing preservatives, similar rates of cataract formation or maturation (4/34 (11.8%) eyes) were observed [25]. Because cataract maturation was observed most often in eyes with moderate pre-existing cataracts, we speculated that the preservatives in Genta 100 may have contributed to the accelerated cataract maturation in these cases. In order to minimize this risk, we switched to PFG solution after making this observation. After switching to PFG, only a single cataract progressed from immature to mature (1/52 eyes, 1.9%). Although the exact risk of cataract maturation associated with PFG IVGI is unknown, it appears that utilization of a PFG solution may help to minimize the actual risk of developing this blinding complication post-IVGI.

## Conclusion

With less than 9% of the horses in the present study developing recurrent or persistent inflammation, less than 9% with cataract maturation and less than 6% with retinal degeneration, IVGI was associated with a lower level of complications compared with medical therapy [3, 17, 18] and other commonly implemented surgical treatment options for ERU (CSI placement and PPV) [10–13]. The ability of low-dose IVGI with 4 mg gentamicin (especially PFG) to suppress active inflammation in various types and stages of equine uveitis in the present study despite the *Leptospira* status of the eye, adds another treatment option in the management of a severely debilitating and vision threatening disease.

## Methods

### Case selection

Complete initial and all follow-up ophthalmic examinations were performed by a board-certified veterinary ophthalmologist (RJM) between January 2013 through June 2016 in south-east Germany. Horses presenting with signs of active or chronic uveitis and a history of recurrences were included in the study. Signs associated with recurrent or persistent uveitis included, but were not limited to, blepharospasm, epiphora, keratic precipitates (KP), aqueous flare, fibrin in the anterior chamber (AC), hyphema, miosis, corpora nigra atrophy or degeneration, iris hyper- or depigmentation, equatorial vesicular cataracts, posterior lens capsule adhesions or opacifications, vitreous body opacifications, and retinal detachment. Horses with uveitis resulting from putative trauma, secondary to infectious corneal diseases, or following intraocular surgery, were excluded.

Owners were educated on the various medical and surgical (i.e., PPV, CSI, and intravitreal injections) treatment options, and risks associated with each option. Client consent to perform the IVGI was obtained following an in-depth discussion of potential complications including failure of the selected treatment option to control the disease, resulting in persistent/ recurrent inflammation with progression of ocular signs, and potential cataract maturation or development and retinal degeneration or detachment.

### Examination

Complete ophthalmic examinations were performed on initial presentation, and on each subsequent follow-up examination, and consisted of a subjective clinical vision assessment (menace response) and neuro-ophthalmic evaluation (dazzle, and pupillary light reflexes (PLR)), slit lamp bio microscopy (Kowa SL-15)^,^^1^ indirect ophthalmoscopy (Keeler Vantage),^2^ rebound tonometry (TonoVet),^3^ external ocular fluorescein dye application (Fluoreszein SE Thilo),^4^ and color (Nikon D300s)^5^ and infrared (Nikon D200)^5^ (sensor conversion)^6^ digital imaging. Aqueous flare was graded as follows: 0 (none), 1 (faint), 2 (moderate), 3 (severe) or 4 (blood or fibrin present in the anterior chamber) [30]. Fundus images (Kowa Genesis or ClearView)^1,^^7^ were obtained in horses with posterior segment abnormalities, when possible.

### Categorization of uveitis

**Fig. 4:**
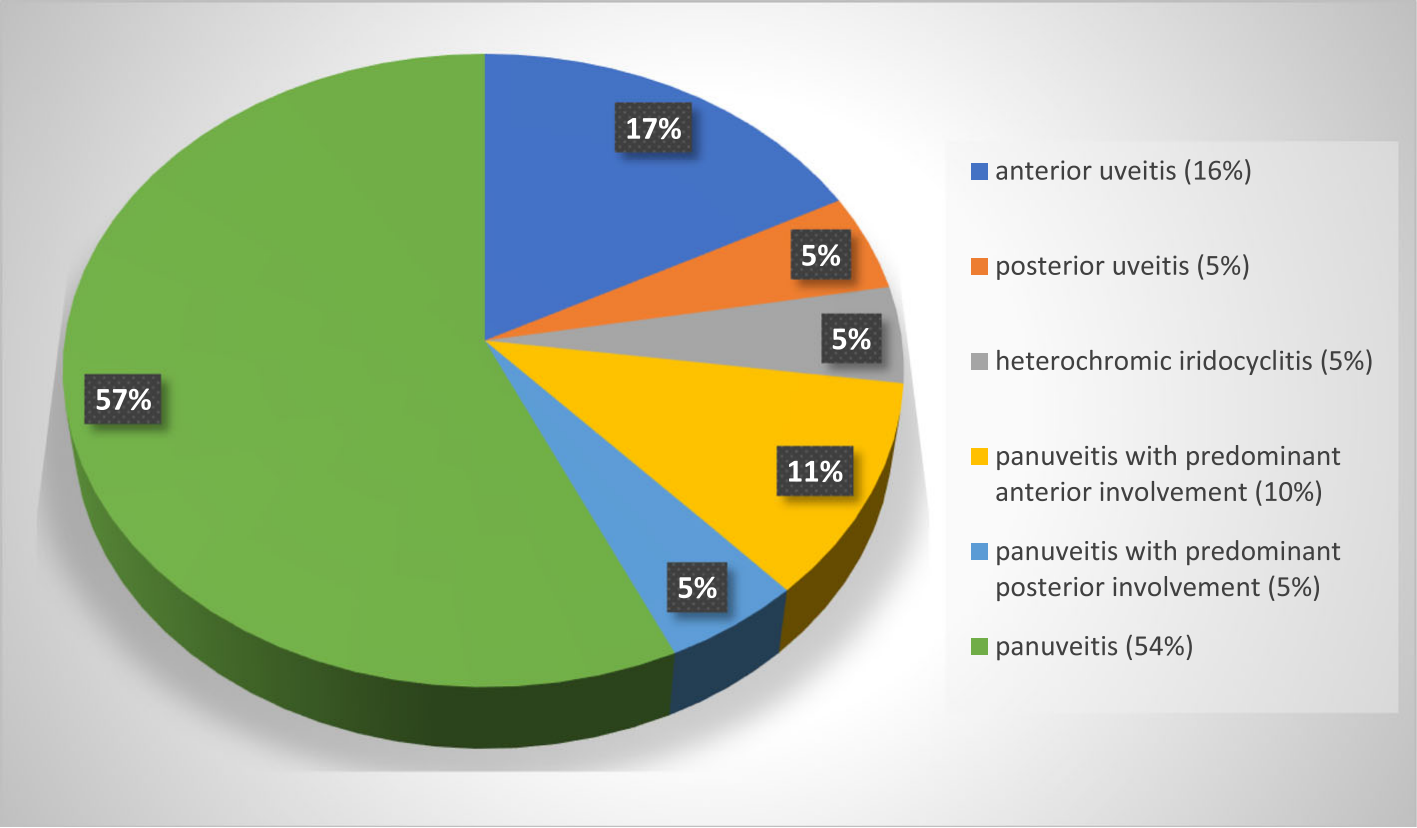
Distribution of clinical signs of uveitis

For statistical purposes, each case was diagnosed with one of the following: 1. Panuveitis (global uveal inflammation with equal distribution of clinical signs between the anterior and posterior segment); (Figs. 2 and 4; 2. Panuveitis with predominant anterior segment involvement; 3. Panuveitis with predominant posterior segment involvement; 4. Anterior uveitis; (Fig. 3); 5. Posterior uveitis; and 6. Heterochromic iridocyclitis with secondary keratitis (HIK), a recently described, specific form of idiopathic anterior uveitis (iridocyclitis) and corneal endothelial inflammation associated with iris pigment dispersion and retro-corneal fibrous membrane formation [2, 3, 8, 31].

Each case was further categorized as **“acute”** (active inflammation without overt signs of chronicity), **“chronic”** (signs of chronicity but no signs of active inflammation) or **“acute/chronic”** (acute onset of inflammation associated with chronic inflammation). **“Recurrent uveitis”** was diagnosed when at least 2 episodes of recurrent inflammation occurred despite appropriate medical therapy leading to a period of quiescence following cessation of medical therapy. **“Persistent inflammation”** was diagnosed when an initial or recurrent bout of inflammation remained actively inflamed for a minimum of four weeks despite aggressive and appropriate medical or surgical therapy (Fig. 4).

The subjective vision status prior to, and following, IVGI was graded as “***good”*** (positive menace, dazzle, direct and indirect PLR, with no evidence of obvious visual field impairment due to corneal edema, hyphema, hypopyon, fibrin in the anterior chamber, miosis, synechia, lens opacities/cataracts, vitreal degeneration, fundus abnormalities), “***reduced”*** (positive menace, dazzle, direct and indirect PLR with some evidence of visual field impairment due to the abnormalities listed above) and “***poor”*** (negative or positive menace, dazzle and direct and indirect PLR with obvious evidence of visual field impairment (late immature to mature cataracts), retinal degeneration/ detachment, or phthisis bulbi).

### Sedation, intravitreal gentamicin injection and aqueous paracentesis

A general physical examination including auscultation and body temperature measurement was performed prior to sedation with a combination of detomidine hydrochloride (Domosedan, 0.01–0.02 mg/kg bwt i.v.)^8^ and butorphanol (Alvegesic, 0.005–0.01 mg/kg bwt i.v.)^9^ intravenous and intramuscular bolus (0.02–0.04 mg/kg bwt i.m.) injections. Blood (serum and EDTA) was drawn and submitted for a complete blood count and chemistry panel. A microscopic agglutination test (MAT) for *Leptospira* antibodies was also routinely performed [32]. A total of nine serovars (*L. bratislava,*^10^ *L. icterohaemorrhagiae/copenhageni*^10^, *L. australis*^10^, *L. pomona*^10^, *L. grippotyphosa*^10^, *L. autumnalis*^10^, *L.canicola,*^11^ *L. saxkoebing*^11^, *L.sejroe*^11^) were evaluated^10,11^ .

Following sedation, the horse’s head was positioned atop a pair of pads placed on a mobile cart to increase stability. Local akinesia and analgesia (palpebral and the frontal nerve blocks) was achieved using 2% mepivacaine (Scandicain 2%)^12^ [33]. Topical anesthetic ophthalmic solution (proparacaine HCL 0.5%)^13^ was applied as needed. The conjunctival fornices were irrigated with 1.0 ml of a dilute baby shampoo solution^14^ (1 ml of baby shampoo in 1 l of balanced saline solution), 1.0 ml of a 1.0% dilute iodine solution,^15^ and 1.0 ml of balanced saline solution (Acrisol)^4^ [34].

Dorsal globe exposure was facilitated with either a Desmarres^16^ or prototype eyelid retractor,^17^ and further enhanced by rotating the horse’s head away from the examiner to exaggerate ventral globe rotation. The first 34 horses were treated with a 4 mg injectable gentamicin solution containing preservatives (Genta100; 100 mg/ml)^9^. All additional horses (*n* = 52) were treated with preservative-free gentamicin (PFG) (Gentamicin-ratiopharm, 160 mg/2 ml SF).^18^ Undiluted gentamicin (0.04 ml Genta100 or 0.05 ml PFG) ^9,18^ was drawn up in a 30- gauge needle/syringe combination (12 mm length, 1.0 ml insulin syringe), and the IVGI was performed using headloupes (magnification) (Eschenbach MaxView with LED light source or headloupes with a separate head-mounted light source (ML4-LED))^19^^,^^20^^,.^^21^ The injection site was 10 mm posterior to the limbus at 12 o’clock. Injection was facilitated by applying gentle but steady pressure while slowly and deliberately rotating the needle in a clockwise manner with the needle directed toward the optic nerve head to avoid inadvertent contact with the lens. Aqueous paracentesis was then performed using a second insulin syringe at either the 11:00 o’clock (right eye, oculus dexter, OD) or 1:00 o’clock (left eye, oculus sinister, OS) positions. A total volume of 1 ml aqueous humor was aspirated. Aqueous humor and serum samples were refrigerated prior to transport to the laboratory. MAT tests for *Leptospira* titers were performed with the serum and aqueous humor, and real-time PCR was used for the detection of *Leptospira* DNA in the aqueous humor [35]. With the help of the C-value (dividing the aqueous humor *Leptospira* antibody titer by the serum *Leptospira* antibody titer), eyes were categorized into ***Leptospira*** **“positive”** (C-value greater than 3), **“*Leptospira*** **suspicious”** (C-value between 1 and 3) and **“*Leptospira*** **negative”** (C-value of 0) for statistical evaluation [27, 36].

### Post-injection therapy

Post IVGI medical therapy consisted of topical antibiotics (Ofloxacin)^22^ q8h for one week, and topical corticosteroids (Prednisolone acetate)^23^ or nonsteroidal anti-inflammatory drugs (NSAIDs) (Bromfenac)^22^ that were gradually tapered over the course of 4–8 weeks based on each horse’s individual response to therapy. Either 1% tropicamide^24^ or 1% atropine^25^ were applied topically for a variable duration to maintain or achieve mydriasis and to stabilize the blood aqueous barrier. Systemic NSAIDs (flunixin-meglumine, 0.55 mg/kg, p.o., q12h)^9^ were also administered per os and gradually tapered-off over the course of 7 to 14 days. A prophylactic dose of 37% omeprazole (Gastrogard, 2 mg/kg p.o.)^26^ was routinely administered orally, once daily while using systemic NSAIDs.

### Follow-up examination

Following IVGI all eyes were immediately examined for the presence of peri-injection complications (subconjunctival or intracameral hemorrhage), and re-examined within 24 h. Horses were monitored weekly for the first month or until medications were discontinued. Subsequent follow-up examinations were spaced further apart based on the horse’s individual response to treatment. Inflammation was considered controlled if no signs of recurrent or persistent uveitis, independent of complications, were identified at any follow-up examination after medications had been discontinued. Particular attention was paid at all times to the possible development of post-injection complications (cataract formation/maturation, retinal degeneration).

### Data analysis

All 86 eyes were evaluated for peri-injection complications (subconjunctival and intracameral hemorrhage), but only those with a minimum follow-up period of 30 days (59 eyes) were included in the post-injection statistical data evaluation. These latter eyes were monitored for signs of recurrent or persistent inflammation, as well as for the presence of additional complications or sequelae (e.g., cataract formation/progression and retinal degeneration). Correlation between the outcome (controlled, recurrent, or persistent inflammation), post-injection complications (no complications, cataract formation or maturation, and retinal degeneration), and possible influencing factors (breed, coat color, gender, clinical diagnosis with chronic or acute and recurrent or persistent, Leptospira status of the eye, Leptospira PCR, individual C-values for each *Leptospira* serovar, the combined C-value, the *Leptospira* serum and aqueous humor titer, response to topical medication prior to IVGI, severity of signs, frequencies of recurrence, gentamicin with preservatives, PFG, visual reflex tests, vision status prior to IVGI, miosis before IVGI, subconjunctival hemorrhage, intracameral hemorrhage and glaucoma prior to IVGI) were determined using a Pearson’s chi-squared test or a Fisher’s exact test. To avoid same-animal correlation, a single eye per horse was randomly selected for inclusion in the study from those receiving bilateral IVGI. Differences with *P* ≤ 0.05 were considered significant. Results were calculated using computerized statistical software (IBM SPSS 23.0).^27^

## Abbreviations

AH: Aqueous humor
CSI: Cyclosporine implant
ERG: Electroretinogram
ERU: Equine recurrent uveitis
HIK: Heterochromic iridocyclitis and keratitis
IVGI: Intravitreal gentamicin injection
MAT: Microscopic agglutination test
NSAID: Nonsteroidal anti-inflammatory drug
OCT: Optical coherence tomography
PFG: Preservative-free gentamicin
PLR: Pupillary light reflex
PPV: Pars plana vitrectomy
S: Serum

## Footnotes

1 Kowa Company Ltd., Tokyo, Japan

2 Keeler Instruments Inc., Broomall, Pennsylvania, USA

3 Icare, Oy, Vantaa, Finland

4 Alcon, Pharma GmbH, Freiburg im Breisgau, Germany

5 Nikon GmbH, Düsseldorf, Germany

6 Life Pixel, Mukilteo, Washington, USA

7 Kruuse A/S, Langeskov, Denmark

8 Pfizer Animal Health, Exton, Pennsylvania, USA

9 CP-Pharma, Handelsges. mbH, Burdorf Germany

10 Idexx Laboratories, Inc., Ludwigsburg, Germany

11 Laboklin GmbH und Co.KG, Bad Kissingen, Germany

12 AstraZeneca GmbH, Wedel, Germany

13 Ursapharm Azneimittel GmbH, Saarbrücken, Saarland

14 Johnson and Johnson GmbH, Neuss Germany

15 aniMedica GmbH, Senden-Bösensell, Germany

16 B.Braun Welsungen AG, Welsungen, Germany

17 Eickemeyer-Medizintechnik für Tierärzte KG, Tuttlingen, Germany

18 Ratiopharm GmbH, Ulm, Germany

19 Eschenbach Optik GmbH, Nürnberg, Germany

20 Zeiss, Jena, Germany

21 Heine Optotechnik, Herrsching, Germany

22 Bausch and Lomb GmbH., Berlin, Germany

23 Dr. Winzer GmbH, Berlin, Germany

24 Pharma Stulln GmbH, Stulln, Germany

25 Römhild pharmacy, Diessen, Germany

26 Merial GmbH, Hallbergmoos, Germany

27 IBM, Armonk, New York, USA
